# Acceptability and Effectiveness of NHS-Recommended e-Therapies for Depression, Anxiety, and Stress: Meta-Analysis

**DOI:** 10.2196/17049

**Published:** 2020-10-28

**Authors:** Melanie Simmonds-Buckley, Matthew Russell Bennion, Stephen Kellett, Abigail Millings, Gillian E Hardy, Roger K Moore

**Affiliations:** 1 Department of Psychology University of Sheffield Sheffield United Kingdom; 2 Department of Computer Science The University of Sheffield Sheffield United Kingdom; 3 Sheffield Health and Social Care NHS Foundation Trust Sheffield United Kingdom; 4 Centre for Behavioural Science and Applied Psychology Sheffield Hallam University Sheffield United Kingdom

**Keywords:** e-therapy, anxiety, depression, treatment effectiveness, National Health Service, meta-analysis, mobile phone

## Abstract

**Background:**

There is a disconnect between the ability to swiftly develop e-therapies for the treatment of depression, anxiety, and stress, and the scrupulous evaluation of their clinical utility. This creates a risk that the e-therapies routinely provided within publicly funded psychological health care have evaded appropriate rigorous evaluation in their development.

**Objective:**

This study aims to conduct a meta-analytic review of the gold standard evidence of the acceptability and clinical effectiveness of e-therapies recommended for use in the National Health Service (NHS) in the United Kingdom.

**Methods:**

Systematic searches identified appropriate randomized controlled trials (RCTs). Depression, anxiety, and stress outcomes at the end of treatment and follow-up were synthesized using a random-effects meta-analysis. The grading of recommendations assessment, development, and evaluation approach was used to assess the quality of each meta-analytic comparison. Moderators of treatment effect were examined using subgroup and meta-regression analysis. Dropout rates for e-therapies (as a proxy for acceptability) were compared against controls.

**Results:**

A total of 24 studies evaluating 7 of 48 NHS-recommended e-therapies were qualitatively and quantitatively synthesized. Depression, anxiety, and stress outcomes for e-therapies were superior to controls (depression: standardized mean difference [SMD] 0.38, 95% CI 0.24 to 0.52, N=7075; anxiety and stress: SMD 0.43, 95% CI 0.24 to 0.63, n=4863), and these small effects were maintained at follow-up. Average dropout rates for e-therapies (31%, SD 17.35) were significantly higher than those of controls (17%, SD 13.31). Limited moderators of the treatment effect were found.

**Conclusions:**

Many NHS-recommended e-therapies have not been through an RCT-style evaluation. The e-therapies that have been appropriately evaluated generate small but significant, durable, beneficial treatment effects.

**Trial Registration:**

International Prospective Register of Systematic Reviews (PROSPERO) registration CRD42019130184; https://www.crd.york.ac.uk/prospero/display_record.php?RecordID=130184

## Introduction

The potential contribution of digital technology in enabling access to evidenced-based psychological care for mental health problems is high on national and international research, policy, commissioning, and service management agendas [1]. In modern life, as digital tools (eg, mobile phones, tablets, laptops, and wearable devices) have become ubiquitous, psychological interventions delivered by such devices (ie, e-therapies) offer greater convenience and enable constant access to treatment compared with traditional face-to-face therapy with health professionals [2]. The increasing demand for primary care psychological services globally has provided the context within which e-therapies have been integrated into the offer of a suite of *low-intensity (LI)* psychological interventions [3], often delivered within stepped-care systems [4,5]. Although technological innovation in methods of treatment delivery usefully expands availability, it also creates the risk of commercial promotion and availability of ineffective or possibly harmful psychological interventions [6]. Therefore, commissioners, clinicians, and patients need access to reliable and contemporary guidance regarding the empirical status and clinical utility of e-therapies.

The potential organizational, therapeutic, and health economic benefits of e‑therapies initially triggered a global wave of investment and interest [7]. In the United Kingdom, for example, the National Health Service (NHS) Commissioning Board launched the NHS Health Apps Library in March 2013 and NHS Mental Health Apps Library in March 2015. However, the libraries were removed in 2015 after questions were raised concerning e-therapy data security governance [8] and clinical effectiveness [9]. NHS England launched 2 new digital platforms in April 2017, a new beta of the NHS Digital Apps Library and a mobile health space, in an effort to close the gap between e-therapy development and thorough evaluation. Before the removal of the initial NHS App Libraries, a list of 48 NHS-recommended e-therapies was compiled for the National Institute for Health and Care Excellence (NICE) assessment of digitally enabled psychological therapies for use in Improving Access to Psychological Therapies (IAPT) services [10]. A recent quality assessment of the development process of NHS-recommended e-therapies strongly advocated developers to routinely adopt clinical trial methods to test acceptability and efficacy of e-therapies before wider dissemination [11]. NICE has also recently published an evidence standards framework for e-therapies providing guidance concerning efficacy and effectiveness standards [12].

This review aims to quantitatively synthesize the evidence base of e-therapies recommended for use in the NHS for depression, anxiety, and stress in adults to better inform the commissioning and use of e-therapies in clinical services. It was relevant to restrict this review to adults as the NHS-recommended e-therapies are intended for adults. Previously, an individual participant meta-analysis of the e-therapy clinical trial evidence base for depression showed that e-therapy was significantly more effective than controls [13], and there is clinical trial evidence for the efficacy of e-therapy as a treatment for anxiety [14]. This study had 3 aims. First, we sought to quantify the effect of NHS-recommended e-therapies (ie, the 48 e-therapies identified by Bennion et al [10]), as no previous specific meta-analysis of the efficacy of NHS-recommended e-therapies has been attempted. As randomized controlled trials (RCTs) are viewed as the *gold standard evaluation* [15], we sought to only use RCT studies to increase the quality of the meta-analysis. Second because e-therapies are criticized for generating high dropout rates [16], we sought to compare dropout rates in contrast to controls to appraise acceptability. Finally, we sought to investigate the impact of potential moderating factors (eg, gender, age, severity, treatment approach, treatment duration, setting, focus problem, and risk of bias) on e-therapy outcomes via subgroup and meta-regression analyses.

## Methods

The review was registered on the International Prospective Register of Systematic Reviews (PROSPERO; CRD42019130184). The PRISMA (Preferred Reporting Guidelines for Systematic Reviews and Meta-Analyses) are used throughout [17].

### Study Selection

A 3-stage search strategy was developed to identify RCTs evaluating all of the e-therapies recommended by the NHS for the treatment of depression, anxiety, and stress. First, each of the 48 NHS-recommended e-therapies identified by Bennion et al [10] was used to determine those e-therapies to be included in the search strategy. The name of each e-therapy and its platform type (website or app) were combined to develop a series of search terms (eg, “Beating the Blues” AND “Website”) [18]. Electronic searches were conducted using PsycINFO, Web of Science, and PubMed databases to identify relevant e-therapy outcome studies published up until April 2019 (date of final search was April 11, 2019; see Multimedia Appendix 1 for an example search strategy). Second, reference lists of identified studies and previous e-therapy reviews were also searched. Third, as many e-therapies are not developed under their commercial name, a survey was disseminated to the 48 app developers of the identified NHS-recommended e-therapies to identify additional gray literature not captured by the terms used in the database searches [11]. This process was to supplement the identification of all studies associated with any one e-therapy, even when the commercial name was not used in the reporting. A total of 36 out of 48 (75%) app developers responded to the survey, and the full process was reported by Bennion et al [11]. Titles and abstracts were screened initially (MB), with the full texts of identified studies then screened against inclusion and exclusion eligibility criteria (MB). Queries regarding study eligibility were resolved through discussion among reviewers (MB, SK, and AM).

### Eligibility Criteria

Studies were included if the web-based or smartphone app intervention used was one of the 48 NHS-recommended e-therapies [10] for depression, anxiety, and stress; therefore, all studies of other types of e-therapies and for other clinical conditions were excluded. Studies were eligible for inclusion if, and only if, they used an RCT design to examine the efficacy of e-therapy with an adult population (ie, aged >18 years). To be included, the developer of the e-therapy had to be locatable via a Google search when entering the app name as the search term, and the app had to reference the targeted condition (ie, depression, anxiety, or stress) in its marketing literature or be based on a therapeutic tool known to benefit the targeted condition. Posttreatment outcomes were required to have been assessed using a validated measure of anxiety and/or depression symptoms. Comparators included any *control condition,* comprising a wait list or no treatment, placebo or attention-control activity, or treatment as usual (TAU). Only English language articles were included.

### Outcomes

The 2 main outcomes of interest were participant-reported outcomes of (1) depression and/or (2) anxiety and stress taken at posttreatment and at follow-up (where available, to assess the durability of e-therapy effectiveness). Where multiple measures of one outcome were used (ie, 2 measures of depression), the most frequently used measure across the included studies was prioritized. Therefore, each study only contributed one effect size per outcome. Dropout (as a proxy for acceptability) was classified as the percentage of e-therapy and comparator condition noncompleters, as determined by the definition applied in the original study.

### Data Extraction

A priority data extraction tool was designed for the purpose of the review. MB extracted data from the original studies and then reviewers (SK and AM) independently verified the findings. Data were coded according to the following criteria: (1) *study information*—sample size, trial design, context, comparator type, study length, analytic approach (intention to treat [ITT] or completers), and trial quality; (2) *participant characteristics*—mean age, percentage of males, population sample, presenting problem, and diagnostic information or relevant inclusion criteria; (3) *outcome characteristics*—outcome measure and, if applicable, length of follow-up; and (4) *intervention features*—e-therapy program, regularity of instructed use, duration, intervention component details of the comparator condition, and self-help typology. The self-help typology for each e-therapy was coded based on the framework by Newman et al [19]: minimal contact therapy, predominantly self-help, predominantly therapist-administered treatment, or self-administered therapy. This was selected to provide an assessment of the level and extent of therapist support within the e-therapies. Outcome data on depression, anxiety, and stress symptoms and dropout rates were extracted at treatment completion and follow-up (ie, at 6 months or the closest assessment point available).

### Study and Evidence Quality

The Cochrane risk of bias tool [20] was used to assess the methodological quality of the original studies using the Cochrane Review Manager (RevMan) program [21]. All included studies were assessed on 7 elements: (1) randomization, (2) allocation concealment, (3) blinding of participants and personnel, (4) blinding of outcome assessment, (5) data attrition, (6) selective outcome reporting, and (7) other threats to validity. Elements were rated as having low risk, unclear, or high risk of bias. One rater assessed all the included studies, with all studies double rated by 2 other raters (rater 1 assessed 63% [15/24] and rater 2 assessed 37% [9/24]). Cohen kappa coefficient (*k*) was used to assess the interrater agreement on risk of bias overall scores between the primary rater and 2 second raters [22], and these were interpreted using the Landis and Koch [23] categories: <0 as indicating no agreement, 0 to 0.20 as slight, 0.21 to 0.40 as fair, 0.41 to 0.60 as moderate, 0.61 to 0.80 as substantial, and 0.81 to 1 as almost perfect agreement. There was substantial agreement between the primary rater and rater 1 (*k*=.63) and moderate agreement between the primary rater and rater 2 (*k*=.54). Any differences in rating were discussed by the raters to reach a consensus on the overall risk of bias rating for each included study. The grading of recommendations assessment, development, and evaluation (GRADE) approach was used to rate the quality of the evidence included in each meta-analysis conducted [24]. The quality of evidence was assessed on 5 domains: (1) risk of bias in the individual included studies, (2) publication bias, (3) inconsistency, (4) imprecision, and (5) indirectness of treatment estimate effects. The meta-analysis was graded by 2 reviewers (SK and MS) and a consensus agreed (rated as high, moderate, low, or very low quality).

### Effect Sizes

Standardized mean differences (SMDs) were used to assess differences in outcome between e-therapy and the comparator conditions at posttreatment and follow-up. SMDs were computed by calculating Cohen *d* (mean outcome score of the comparator condition subtracted from the mean outcome score of the e-therapy and dividing by the pooled standard deviation). Where available, effect sizes were computed using ITT outcome data. To account for potential biases in studies with small sample sizes, SMDs were converted to Hedges *g* using the *J* adjustment [25]. Effect sizes were calculated so that a beneficial effect of e-therapy was represented by a positive SMD and vice versa. Interpretations of effect size magnitude were classified as 0.20 to 0.49=small, 0.50 to 0.79 = medium, and >0.80=large [26]. When studies had multiple treatment arms delivering e-therapies that could be considered comparable (ie, the same e-therapy with different component combinations, such as reminders and telephone support), the data were collapsed into a single group using Cochrane guidelines [20]. When studies had multiple treatment arms that could not be collapsed (ie, three-arm trial comparing 2 different types of recommended e-therapy to a control), the treatment arms were included independently. The sample size of the shared comparator condition was split evenly across independent treatment arm comparisons to avoid participant data being included twice.

### Data Synthesis

Meta-Essentials workbooks were used to synthesize e-therapy treatment effects in a random-effects meta-analysis to account for the extent of expected study heterogeneity [27]. Individual study effect sizes were weighted using the inverse of the variance to produce overall pooled treatment effect estimates and 95% CIs. The threshold for statistical significance was set at an αvalue of .05. The *I^2^* statistic was employed as an indicator of the percentage of between-study heterogeneity, whereas the *Q* statistic provided a test of the statistical significance of the presence of study variation. Thresholds of heterogeneity were interpreted as <40% may not be relevant, 30% to 60% representing moderate heterogeneity, 50% to 90% representing substantial heterogeneity, and 75% to 100% representing considerable heterogeneity [28]. As recommended by Cochrane, the magnitude and direction of effect sizes were used to interpret the implications of *I^2^* percentages. The overall pooled effect sizes of e-therapy were translated into *numbers needed to treat* (NNTs) [29]. NNT is an approximation of how many patients would need treatment with e-therapy to generate an additional outcome of benefit when compared with another intervention (ie, the comparator condition). A Mann-Whitney U test was used to assess for differences in dropout rates between e-therapy and controls.

### Moderator and Sensitivity Analyses

Preplanned random-effects moderator analyses were performed using the Meta-Essentials workbooks to evaluate between-study variation in treatment effects in posttreatment comparisons with a minimum of 10 studies [20]. Moderators were selected based on methodological, clinical, and intervention features that were likely to vary between studies. Meta-regressions were applied to 5 continuous variables: mean age, mean number of sessions completed, percentage of males, baseline symptom severity (standardized Z scores), and risk of bias (number of items meeting criteria for low risk of bias: 0-7). Subgroup analyses were applied to 6 categorical variables: 4 of them were specified a priori (control type, e-therapy type, self-help typology, and recruitment setting) and 2 were conducted post hoc (focus problem and analysis method). Owing to multiple testing, the αthreshold for significance of the meta-regression beta-coefficients and the between-subgroup differences was lowered to *P*<.01. A series of sensitivity analyses were performed to assess the impact of outliers on the pooled effect sizes (with extreme outliers removed) and to further explore treatment effect durability (comparisons of follow-up effects separately at short-term [1-2 months], medium-term [6 months], and long-term [>8 months] follow-up).

### Publication Bias

Several methods were employed to assess for the presence of publication bias in the posttreatment comparisons that had a sufficient number of studies (k>10). Visual inspection of the asymmetry of a funnel plot (SE plotted against effect sizes) gave an indication of the extent of potential publication bias, whereas the accompanying Trim and Fill imputation [30] accounted for any reporting bias to provide an adjusted treatment estimate. Finally, additional statistical testing of asymmetrical study distribution was undertaken using Egger regression [31].

## Results

### Study Selection

**Figure 1:**
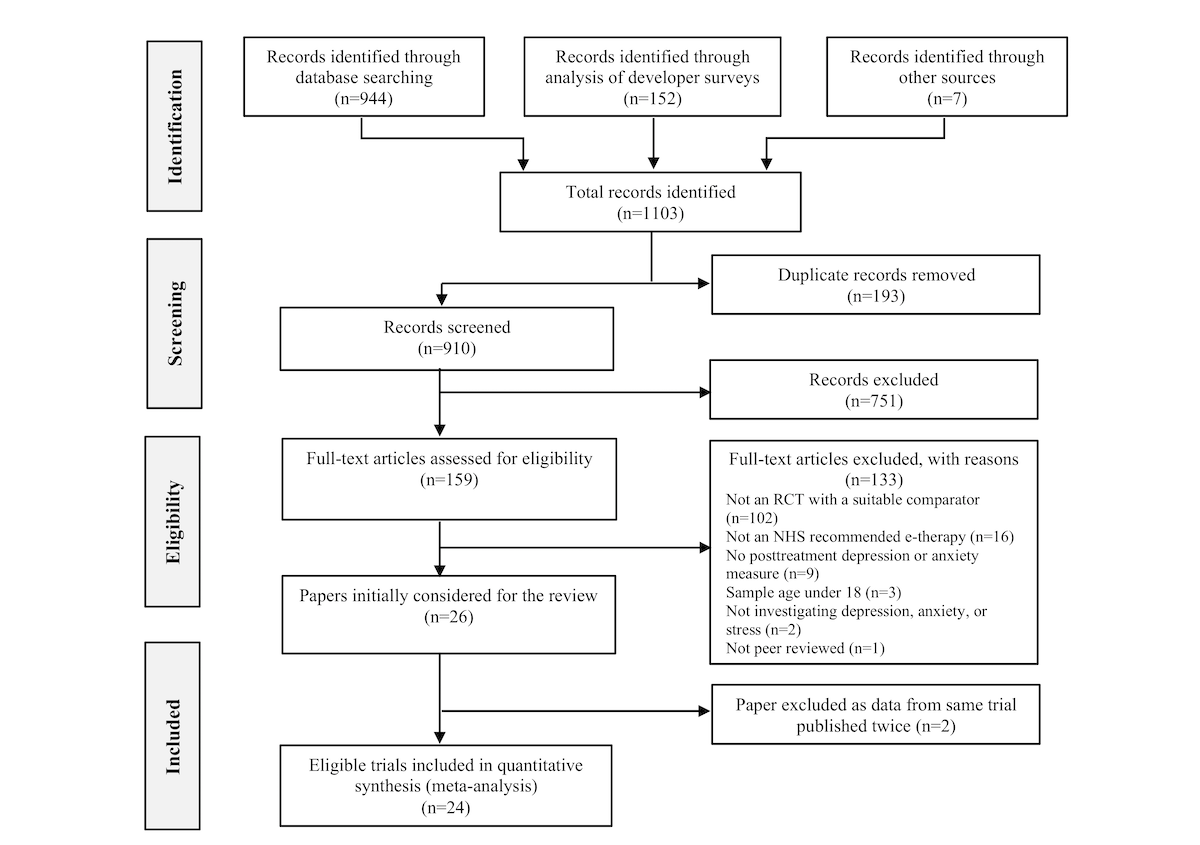
PRISMA (Preferred Reporting Guidelines for Systematic Reviews and Meta-Analyses) flowchart of study selection. NHS: National Health Service; RCT: randomized controlled trial.

The electronic searches returned a total of 944 records. This was combined with the 152 records collected by surveying app developers and 7 records from a manual reference list and review searches, giving a combined total of 1103 records (Figure 1). Duplicates were removed, leaving a total of 910 records to be screened. After excluding records that did not meet the inclusion criteria based on abstracts, 159 full-text articles were retrieved and assessed. Overall, 26 trials were considered eligible, and 2 were excluded because they contained duplicate data from another trial. Thus, a total of 24 studies that tested the efficacy of 7 of the 48 NHS-recommended e‑therapies (Beating the Blues, FearFighter, MoodGYM, IESO, Headspace, Silver Cloud, and Work Guru) in an RCT design were included in the meta-analysis. Details of the included studies can be found in Multimedia Appendix 2 [32-55].

The risk of bias ratings are presented in Table 1. Of the 24 included studies, quality ranged between 1 and 7 quality items meeting low risk of bias criteria (maximum of 7). The overall study quality was moderate to good, with 13 studies meeting low risk of bias criteria on at least five items. A lack of or unclear blinding of participants and personnel or outcome assessment and incomplete outcome data were the most common reasons for risk of bias. For the most poorly rated item across studies, only 3 trials demonstrated suitable blinding of participants and personnel.

**Table 1:** Risk of bias assessment of the included studies.

Study	Risk of bias items						
	1^a^	2^b^	3^c^	4^d^	5^e^	6^f^	7^g^
Proudfoot et al (2003) [32]	+^h^	+	−^h^	?^h^	+	+	+
Grime (2004) [33]	+	+	?	?	?	?	+
Proudfoot et al (2004) [34]	+	+	−	?	+	+	+
Marks et al (2004) [35]	+	+	−	+	?	+	?
Schneider et al (2005) [36]	+	+	−	+	+	+	+
Mackinnon et al (2008) [37]	?	?	−	?	+	+	+
Kessler et al (2009) [38]	+	+	+	+	+	+	+
Ellis et al (2011) [39]	?	?	−	?	?	?	+
Farrer et al (2011) [40]	+	+	?	?	+	?	+
Høifødt et al (2013) [41]	+	+	−	?	+	+	+
Lintvedt et al (2013) [42]	+	+	−	?	+	+	+
Powell et al (2013) [43]	+	+	−	?	+	+	+
Sethi (2013) [44]	+	+	−	−	+	+	+
Howells et al (2016) [45]	+	+	+	?	−	?	+
Phillips et al (2014) [46]	+	+	+	+	+	+	+
Twomey et al (2014) [47]	+	+	−	−	−	+	?
Gilbody et al (2015) [48]	+	+	−	−	+	+	+
Richards et al (2015) [49]	+	+	?	?	+	+	+
Richards et al (2016) [50]	+	+	?	?	+	+	+
Carolan et al (2017) [51]	+	+	−	?	+	+	+
Flett et al (2018) [52]	+	?	−	?	+	+	+
Forand et al (2018) [53]	+	?	−	−	+	+	+
Bostock et al (2019) [54]	+	?	−	?	+	?	+
Löbner et al (2019) [55]	+	+	−	?	+	+	?

^a^Random sequence generation (selection bias).

^b^Allocation concealment (selection bias).

^c^Blinding of participants and personnel (performance bias).

^d^Blinding of outcome assessment (performance bias).

^e^Incomplete outcome data (attrition bias).

^f^Selective outcome reporting.

^g^Other potential threats to validity.

^h^+=low risk; −=high risk; ?=unclear risk.

### Study Characteristics

Out of the 48 NHS e‑therapies identified by Bennion et al [10], a total of 7 (15%) were based on RCT evidence of efficacy, which comprised 6 web-based e‑therapies and 1 smartphone-based e-therapy (Table 2). MoodGYM was the e-therapy with the greatest degree of evaluation (*k*=11 studies), with 2 of the e-therapies having a single RCT evaluation (ie, Ieso and WorkGuru). All 6 web-based e-therapies had both clinical and academic personnel adding expertise during technological development, but the smartphone-based e-therapy had no clinical or academic personnel being involved in its technological development phase [11]. A summary of e-therapy version numbers used in each study and whether a CONSORT-EHEALTH (Consolidated Standards of Reporting Trials of Electronic and Mobile Health Applications and Online Telehealth) checklist [56] was provided (for studies published post-2011 after the checklist was developed) is reported in Multimedia Appendix 3 [32-55]. Reporting of version numbers was generally inconsistent, meaning establishing whether the e-therapies had been updated between studies was difficult. Beating the Blues had been updated between studies, with version 1.0 used in the early studies (2003-2004) [32,34] and version 2.5 used in the most recent study (2018) [53]. Updates to MoodGYM were unable to be established because of inconsistent reporting of version numbers, but there was an indication that the studies between 2011 and 2018 used version III [41,42,55]. It appeared that Headspace was updated from version 1.0 or above in 2014 to a version equal to or above 2.0 in studies from 2019. Studies of FearFighter, SilverCloud, IESO, and WorkGuru either did not refer to version numbers or were only evaluated in 1 RCT, so updates could not be conclusively determined.

All but one of the‑therapies were based on the cognitive behavioral theory (CBT) [11]. E‑therapy treatments lasted between 10 and 70 days (mean 44.52, SD 16.11), comprising between 3 and 45 sessions (mean 8.37, SD 7.98) lasting 10 to 60 min each (mean 48.21, SD 15.26). The majority of e-therapies were administered weekly (*k*=19), whereas 3 of the trials required daily e-therapy usage (2 trials did not report the instructed frequency of usage). Self-help typology was characterized as self-administered therapy (*k*=7 studies), predominantly self-help (*k*=11 studies), minimal contact therapy (*k*=5 studies), and predominantly therapist-delivered treatment (*k*=1 study).

The control conditions employed in the studies were waitlist or no treatment (*k*=13), TAU (*k*=5), and placebo or attention-control tasks (*k*=9; note: *k*=3 studies had multiple control conditions). TAU comprised usual general practitioner (GP) care, allowing access to any treatment prescribed or referred to by a GP. Placebo or attention-control conditions included depression information websites (eg, Bluepages; *k*=2), online peer support forums (eg, MoodGarden; *k*=1), tracking or structured weekly phone calls (*k*=2), neutral tasks or note-taking organization apps (eg, Catch notes software or Evernote; *k*=2), or online self-relaxation (without exposure, ie, a sham treatment; eg, managing anxiety or de-STRESS; *k*=2). In *k*=12 trials, clinical participants were recruited from primary care (*k*=7), psychiatric outpatients (*k*=2), a university counseling center (*k*=1), public sector employees (*k*=1), and a telephone counseling service (*k*=1). In the remaining 12 trials, community participants were recruited from university students (*k*=3), occupational health attendees (*k*=3), the internet (*k*=2), electoral role (*k* =1), youth center (*k*=1), charity users (*k* =1)*, and treatment-seeking adults (k*=1). Mean ages across the samples ranged from 20 to 45 years (mean 35.71, SD 7.76).

E-therapies were delivered for symptoms of depression (*k*=10), anxiety or panic and phobia (*k*=3), stress (*k*=2), or a combination of anxiety and depression symptoms (*k*=6). Three of the trials did not require participants to have any symptoms or indicators of poor mental health. The Beck Depression Inventory (I or II) was the most commonly used depression outcome measure (*k*=7), followed by the Centre for Epidemiologic Studies Depression Scale (CES-d; *k*=6). The most commonly employed anxiety outcome measures were the Generalized Anxiety Disorder-7 (*k*=4) and the Depression Anxiety Stress Scales—anxiety subscale (*k*=4). Follow-up assessments were conducted in 18 trials (*k*=2 had insufficient data to be included in the follow-up analysis). The duration of follow-up ranged between 1 and 20 months (mean 5 months). Dropout rates ranged from 0% to 64%. The average e-therapy dropout rate was 31% (SD 17.35), and the average dropout rate for controls was 17% (SD 13.31). Therefore, significantly more participants dropped out during e-therapies compared with controls (*U*=181.000; *Z*=−3.026; *P*=.002).

**Table 2:** Types of e-therapies used in included studies.

E-therapy	Number of trials^a^	Delivery platform	Clinical involvement	Academic involvement	Psychological theory or clinical approach used	Evidence of updates between studies
Beating the Blues	5	Web-based	Y^b^	Y	CBT^c^	Yes
Fear Fighter	2	Web-based	Y	Y	CBT	Could not be determined
Headspace	3	Phone-based	N^d^	N	Mindfulness	Yes
IESO	1	Web-based	Y	Y	CBT	N/A^e^
MoodGYM	11	Web-based	Y	Y	CBT	Could not be determined
SilverCloud Health	2	Web-based	Y	Y	CBT	N/A
WorkGuru	1	Web-based	Y	Y	CBT, mindfulness, and PP^f^	N/A

^a^A total of 2 e-therapies were evaluated in one trial; therefore, the total number of trials exceeded the overall number of included studies.

^b^Y: yes.

^c^CBT: cognitive behavioral therapy.

^d^N: no.

^e^N/A: not applicable, as e-therapy content was not assessed in multiple studies.

^f^PP: positive psychology.

### Meta-Analysis of E-Therapy Versus Controls

Meta-analytic comparisons were performed to aggregate the effect of e-therapy vs controls on (1) depression and (2) anxiety and stress symptoms at posttreatment and follow-up. GRADE assessments are reported for each comparison, indicating the quality of evidence. All comparisons were based on RCT evidence so they started as high-quality evidence. Across the meta-analyses, limited issues were found in terms of study limitations or publication bias, but some limitations were found for heterogeneity, treatment comparisons, and imprecision. As a result, the level of evidence was downgraded for all comparisons, with the majority demonstrating moderate quality. Comparisons were downgraded one level specifically due to significant and considerable *I^2^* statistic indicating marked heterogeneity in the original studies, variability in primary outcome measure, differing control groups, and varied effects based on lower and upper bounds of confidence intervals. One comparison was downgraded 2 levels to low-quality evidence because of additional limitations created by the small number of studies restricting subsequent moderator analyses and variability in follow-up time.

#### Effect of E-Therapy on Depression Outcomes

##### Posttreatment and Follow-Up Comparisons

**Figure 2:**
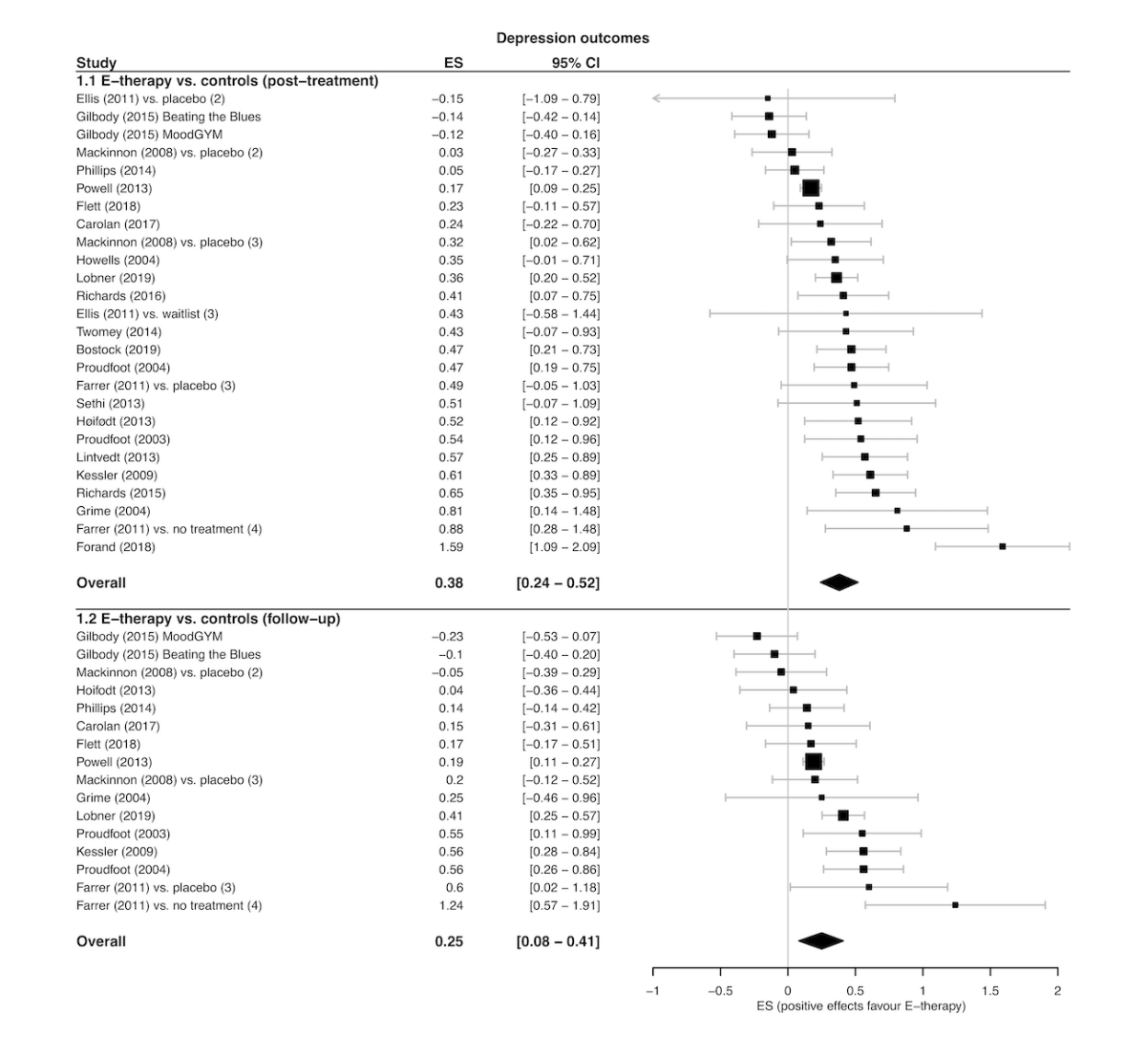
Forest plot of post-treatment and follow-up depression outcome effect sizes (ES) for e-therapy versus controls.

Overall, 26 treatment arm comparisons (extracted from 22 studies) totaling 7075 participants evaluated posttreatment e-therapy depression outcomes in comparison with a control condition (e-therapy, n=3545; control, n=3530). The pooled SMD presented in Figure 2 signified a small, significant treatment effect in favor of greater depression reductions following e-therapy (SMD 0.38; 95% CI 0.24 to 0.52; Z=5.78; *P*<.001; GRADE=moderate). The NNT was 4.72, indicating that for every 5 patients who received e-therapy, there was one additional beneficial depression outcome compared with if they had received a control condition. Between-study variation was significant, indicating substantial heterogeneity between studies (I^2^=73%; 95% CI 60% to 82%; Q=92.30; *P*<.001). Furthermore, 16 follow-up treatment arm comparisons (extracted from 13 studies) provided follow-up data on depression outcomes for e-therapies versus control conditions for 5709 participants (e-therapy, n=2850; control, n=2859). There was a small significant pooled SMD in favor of depression outcomes at follow-up compared with controls (Figure 2; SMD 0.25; 95% CI 0.08 to 0.41; Z=3.23; *P*=.001; NNT=7.12; GRADE=moderate). The between-study variation was significant, indicating moderate-to-substantial heterogeneity (I^2^=69%; 95% CI 48% to 81%; Q=48.11; *P*<.001).

##### Moderator and Sensitivity Analyses

The significant heterogeneity between studies at posttreatment and follow-up was investigated using meta-regression (Table 3) and subgroup moderator analyses (Table 4). Meta-regression analyses found that variations in e-therapy treatment effects were not explained by gender, age, number of sessions, or study quality at posttreatment or follow-up. Although initial depression severity was not significantly associated with effect size at posttreatment, higher levels of depression severity were associated with larger beneficial effects of e-therapy at follow-up. Subgroup analyses showed that variation in posttreatment effect size was associated with the type of control condition (although the effect fell short of significance after accounting for multiple testing). A moderate effect was observed in favor of e-therapy vs wait list controls, whereas the effects for e-therapy compared with placebo conditions and TAU were small. At follow-up, e-therapy effect sizes did not significantly differ according to the control type with e-therapy, showing a small significant beneficial effect compared with placebo and TAU controls and a small nonsignificant effect compared with wait list. Posttreatment and follow-up effects were not significantly affected by the e-therapy type, self-help typology, recruitment setting, focus problem, or analysis method. Substantial significant heterogeneity was evident in approximately half of the subgroups.

**Table 3:** Meta-regression analyses of effect e-therapy vs controls on depression and anxiety outcomes (posttreatment and follow-up).

Time point and outcome, variable					*k* ^a^		B coefficient		95% CI		SE		*P* value^b^		*R*^*2*^ (%)^c^
**Posttreatment**															
	**Depression**														
		Initial severity		26		0.07		−0.06 to 0.21		0.06		.26		4.15	
		Percentage of males		26		-0.01		−0.02 to 0.00		0.01		.09		8.30	
		Mean age (years)		26		0.00		−0.02 to 0.01		0.01		.58		0.95	
		Mean number of sessions completed		17		0.02		0.00 to 0.05		0.01		.08		10.23	
		Risk of bias		26		-0.01		−0.11 to 0.08		0.05		.77		0.28	
**Follow-up^c^**															
	**Depression**														
		Initial severity		16		0.25		0.12 to 0.39		0.06		<.001		53.17	
		Percentage of males		16		-0.01		−0.03 to 0.01		0.01		.13		11.64	
		Mean age (years)		16		0.01		−0.01 to 0.04		0.01		.38		3.88	
		Mean number of sessions completed		11		0.01		−0.06 to 0.08		0.03		.78		0.44	
		Risk of bias		16		0.02		−0.11 to 0.14		0.06		.78		0.40	
**Posttreatment**															
	**Anxiety^d^**														
		Initial severity		17		0.12		−0.07 to 0.31		0.09		.17		8.84	
		Percentage of males		17		-0.01		−0.03 to 0.01		0.01		.24		5.85	
		Mean age (years)		17		-0.01		−0.03 to 0.01		0.01		.43		3.03	
		Mean number of sessions completed		11		0.02		0.00 to 0.05		0.01		.07		23.93	
		Risk of bias		17		-0.01		−0.14 to 0.12		0.06		.85		0.18	

^a^*k*: number of comparisons.

^b^Alpha threshold Bonferroni adjusted to *P*<.01 for multiple testing*.*

^c^Insufficient number of comparisons and limited between-study heterogeneity to warrant moderator analyses of anxiety outcomes at follow-up.

^d^*R^2^*: percentage of variance explained by the moderator.

**Table 4:** Subgroup analysis of effect e-therapy versus controls on depression outcomes (posttreatment and follow-up).

Time point and variable, Subgroup			*k* ^a^	SMD^b^ (Hedges g)^c^	95% CI	I^2^ (%)^d^	*P* value (between subgroups)^e^	*R*^*2*^ (%)^f^	NNT^g^
**Posttreatment**									
	**Control type**								
		Wait list	12	0.54^h^	0.34 to 0.75	79^h^	.02	8.00	3.36
		TAU^i^	7	0.32^h^	0.06 to 0.58	79^h^	—^j^	—	5.58
		Placebo	7	0.20^h^	0.06 to 0.34	2	—	—	8.89
	**E-therapy type**								
		MoodGYM	14	0.29^h^	0.15 to 0.43	57^h^	.30	3.94	6.15
		Beating the Blues	5	0.55^h^	0.00 to 1.10	89^h^	—	—	3.30
		Headspace	3	0.36^h^	0.22 to 0.49	0	—	—	4.97
		Other	4	0.50^h^	0.32 to 0.68	2	—	—	3.61
	**Self-help typology**								
		Self-administered	8	0.30^h^	0.15 to 0.45	65^h^	.08	5.87	5.95
		Predominantly self-help	14	0.39^h^	0.16 to 0.62	76^h^	—	—	4.60
		Minimal contact	3	0.53^h^	0.39 to 0.67	0	—	—	3.42
		Predominantly therapist delivered	1^k^	0.61	—	—	—	—	2.95
	**Setting**								
		Clinical	12	0.39^h^	0.22 to 0.57	68^h^	.91	0.01	4.60
		Community	14	0.38^h^	0.18 to 0.58	76^h^	—	—	4.72
	**Focus problem**								
		Depression	12	0.39^h^	0.13 to 0.64	84^h^	.74	0.79	4.60
		Anxiety or stress	3	0.38^h^	0.25 to 0.52	0	—	—	4.72
		Both	7	0.47^h^	0.29 to 0.65	0	—	—	3.84
	**Analysis method**								
		ITT^l^	9	0.39^h^	0.24 to 0.54	76^h^	.50	0.49	4.60
		Completers	3	0.33^h^	0.21 to 0.44	0	—	—	5.42
**Follow-up**									
	**Control type**								
		Wait list	4	0.29	−0.15 to 0.73	71^h^	.75	1.19	6.15
		TAU	7	0.29^h^	0.03 to 0.54	79^h^	—	—	6.15
		Placebo	5	0.18^h^	0.00 to 0.36	0	—	—	9.87
	**E-therapy type**								
		MoodGYM	9	0.21	−0.01 to 0.43	73^h^	.79	0.96	8.47
		Beating the Blues	4	0.31	−0.03 to 0.64	73^h^	—	—	5.76
		Other	3	0.32^h^	0.05 to 0.59	51	—	—	5.58
	**Self-help typology**								
		Self-administered	4	0.16	−0.10 to 0.41	80^h^	.46	1.29	11.10
		Predominantly self-help	10	0.29^h^	0.07 to 0.51	65^h^	—	—	6.15
		Minimal contact	1^k^	0.04	—	—	—	—	44.32
		Predominantly therapist delivered	1^k^	0.56	—	—	—	—	3.25
	**Setting**								
		Clinical	10	0.33^h^	0.09 to 0.57	77^h^	.13	4.68	5.42
		Community	6	0.14^h^	0.07 to 0.21	0	—	—	12.68
	**Focus problem**								
		Depression	10	0.22	−0.01 to 0.46	77^h^	.07	7.42	8.08
		Anxiety or stress	1^k^	0.15	—	—	—	—	11.83
		Both	3	0.49^h^	0.32 to 0.66	0	—	—	3.69
	**Analysis method**								
		ITT	3	0.27^h^	0.09 to 0.45	71^h^	—	—	6.60
		Completers	1^k^	0.17	—	—	—	—	10.45

^a^*k*: number of comparisons.

^b^SMD: standardized mean difference.

^c^Positive effect size indicates in favor of e-therapy*.*

^d^Significance of associated *Q* statistic.

^e^Alpha threshold Bonferroni adjusted to *P*<.01 for multiple testing*.*

^f^*R^2^*: percentage of variance explained by moderator.

^g^NNT: number needed to treat.

^h^Significant at *P*<.05.

^i^TAU: treatment as usual.

^j^One between-groups *P* value and R^2^ value are provided for each subgroup comparison, reported on the row of the first subgroup category.

^k^Where there is only one comparison within a subgroup, 95% confidence intervals and I^2^ values are not reported.

^l^ITT: intention to treat.

Sensitivity analyses explored the impact of the extreme outliers and length of follow-up on the pooled depression effect sizes. Although the removal of outlier effects resulted in a slight reduction in the effect of e-therapy on depression from 0.38 to 0.34 at posttreatment and from 0.25 to 0.22 at follow-up, outcomes still indicated small, significant benefits of e-therapy compared with controls. E-therapy demonstrated a small, beneficial effect compared with controls at short-term and medium-term follow-up, which diminished at long-term follow-up. The full sensitivity analysis results are reported in Multimedia Appendix 4.

##### Assessment of Publication Bias

**Figure 3:**
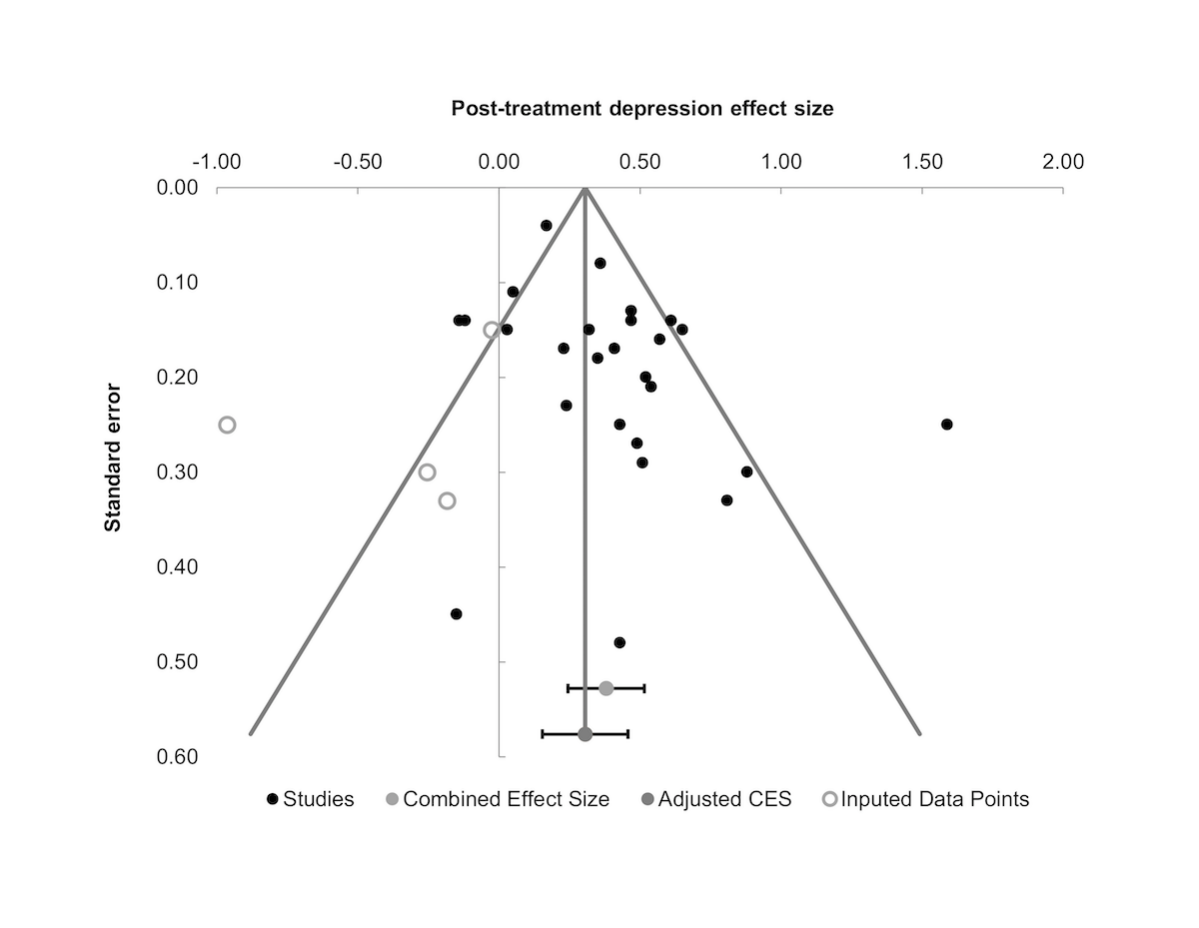
Funnel plot for distribution of studies reporting e-therapy versus controls post-treatment depression outcomes.

Visual inspection of the posttreatment funnel plot (Figure 3) suggested that there was some asymmetry in the distribution of studies, indicating that the smaller included studies were more likely to report larger effects for e-therapy interventions. Trim and fill imputed missing data to represent 4 smaller studies with effects more in favor of controls, producing a slightly reduced adjusted effect size in favor of e-therapy (SMD 0.31; 95% CI 0.15 to 0.46). Statistical testing of publication bias using Egger’s regression did not detect significant asymmetry in the study distribution for posttreatment outcomes (B=−0.15; t_25_=1.49; *P*=.15). Assessment of study distribution for follow-up depression outcomes also did not detect a significant influence of publication bias (B=0.31; t_15_=1.34; *P*=.20). Taken together, the multiple assessments of publication bias suggest a minimal-to-small influence of bias on the overall e-therapy treatment effect for depression outcomes.

#### Effect of E-Therapy on Anxiety and Stress Outcomes

##### Posttreatment and Follow-Up Comparisons

**Figure 4:**
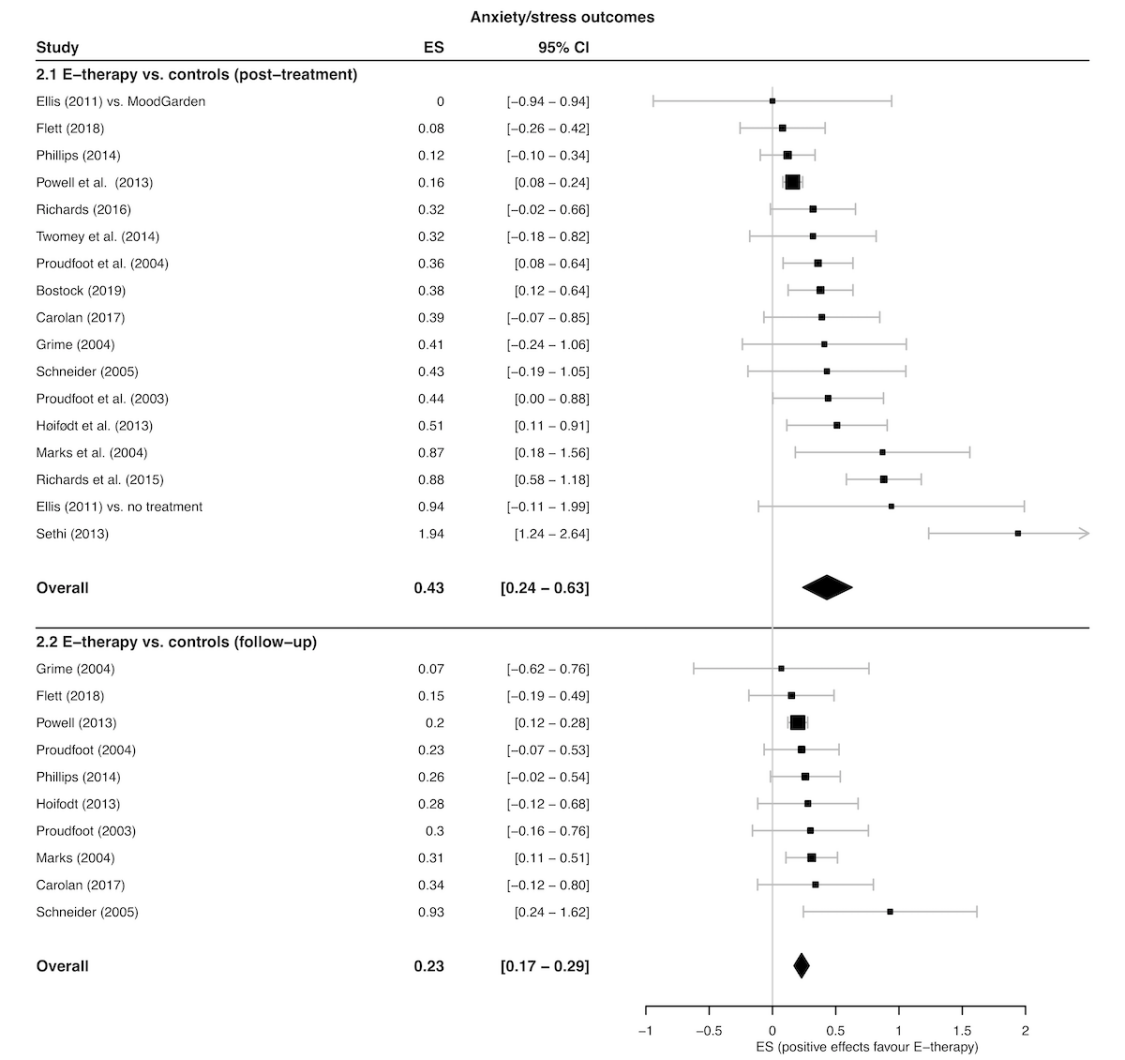
Forest plot of post-treatment and follow-up stress/anxiety outcome effect sizes (ES) for e-therapy versus controls.

Overall, 17 treatment arm comparisons (extracted from 16 studies) totaling 4863 participants evaluated posttreatment e-therapy anxiety and stress outcomes alongside a control condition (e-therapy, n=2443; control, n=2420). The pooled SMD presented in Figure 4 signified a small-to-moderate, significant treatment effect in favor of greater anxiety reductions following e-therapy (SMD=0.43; 95% CI 0.24 to 0.63; Z=4.63; *P*<.001; GRADE=moderate). The NNT was 4.18, indicating that for approximately every 4 patients who received e-therapy, there was one additional beneficial anxiety and stress outcome compared with if they had received a control condition. The between-study variation was significant, indicating substantial heterogeneity (I^2^=73% [95% CI 56% to 83%]; Q=59.13; *P*<.001). Furthermore, 10 studies provided follow-up data on anxiety and stress outcomes for e-therapies vs control conditions for 3983 participants (e-therapy, n=2000; control, n=1983). At follow-up, there was a small, significant pooled SMD in favor of e-therapy compared with controls (Figure 4; SMD=0.23; 95% CI 0.17 to 0.29; Z=8.30; *P*<.001; NNT=7.74; GRADE=low). The between-study variation was minimal and not significant (I^2^=0% [95% CI 0% to 46%]; Q=6.31; *P*=.71).

##### Moderator and Sensitivity Analyses

The significant heterogeneity between studies at posttreatment was investigated with meta-regression (Table 3) and subgroup moderator analyses. Minimal heterogeneity and *an insufficient number of studies (k*<10) negated the need for moderator analysis of follow-up effects. Meta-regression analyses found variations in e-therapy posttreatment anxiety and stress effects were not explained by initial severity, gender, age, number of sessions, or study quality. Subgroup analyses showed that posttreatment effect sizes for anxiety and stress symptoms did not significantly differ for different control conditions. However, e-therapy vs wait list produced a moderate, significant effect compared with the small effects observed for TAU and placebo controls (placebo effect not significant). Posttreatment effects were not significantly affected by the e-therapy type, recruitment setting, focus problem, or analysis method. Self-help typology indicated larger effects were observed for therapies with greater therapist involvement (*P*=.02); however, the effect did not remain significant when applying a Bonferroni correction. Substantial significant heterogeneity was evident in about a quarter of the subgroups.

Sensitivity analyses explored the impact of extreme outliers and length of follow-up on the pooled anxiety and stress effect sizes. Although the removal of outlier effects resulted in a slight reduction in the e-therapy treatment effect on anxiety from 0.43 to 0.37 at posttreatment and from 0.23 to 0.22 at follow-up, the outcomes still indicated small, significant benefits of e-therapy compared with controls. E-therapy demonstrated a small, beneficial effect compared with controls at both short-term and medium-term follow-up (insufficient studies of long-term follow-up were available). The full sensitivity analysis results are reported in Multimedia Appendix 4.

The significant heterogeneity between studies at posttreatment was investigated with subgroup moderator analyses (Table 5)

**Table 5:** Subgroup analysis of effect e-therapy versus controls on anxiety and stress outcomes (posttreatment).

Time point^a^ and variable, Subgroup			*k* ^b^	SMD^c^ (Hedges g)^d^	95% CI	I^2^ (%)^e^	*P* value (between subgroups)^f^	*R*^*2*^ (%)^g^	NNT^h^
**Posttreatment**									
	**Control type**								
		Wait list	9	0.55^i^	0.24 to 0.86	84^i^	.41	2.99	3.04
		TAU^j^	3	0.40^i^	0.35 to 0.45	0	—^k^	—	4.49
		Placebo	5	0.26	−0.02 to 0.55	28	—	—	6.86
	**E-therapy type**								
		MoodGYM	7	0.44^i^	0.01 to 0.86	80^i^	.86	0.50	4.09
		Beating the Blues	3	0.40^i^	0.35 to 0.45	0	—	—	4.49
		Other	7	0.46^i^	0.24 to 0.68	61^i^	—	—	3.92
	**Self-help typology**								
		Self-administered	4	0.23^i^	0.09 to 0.36	8	.02	13.38	7.74
		Predominantly self-help	8	0.47^i^	0.11 to 0.83	74^i^	—	—	3.84
		Minimal contact	5	0.60^i^	0.36 to 0.83	45	—	—	3.04
		Predominantly therapist delivered	0^l^	—	—	—	—	—	—
	**Setting**								
		Clinical	8	0.44^i^	0.33 to 0.54	0	.99	0.00	4.09
		Community	9	0.44^i^	0.10 to 0.78	84^i^	—	—	4.09
	**Focus problem**								
		Depression	3	0.49^i^	0.05 to 0.93	88^i^	.85	0.82	3.69
		Anxiety or stress	5	0.44^i^	0.27 to 0.62	0	—	—	4.09
		Anxiety or depression	7	0.58^i^	0.14 to 1.02	70^i^	—	—	3.14
	**Analysis method**								
		ITT^m^	7	0.47^i^	0.27 to 0.68	75^i^	.06	5.76	3.84
		Completers	2	0.18	−0.05 to 0.42	0	—	—	9.87

^a^Insufficient number of comparisons and limited between-study heterogeneity to warrant moderator analyses of anxiety outcomes at follow-up.

^b^*k*: number of comparisons.

^c^SMD: standardized mean difference.

^d^Positive effect size indicates in favor of e-therapy*.*

^e^Significance of associated *Q* statistic.

^f^Alpha threshold Bonferroni adjusted to *P*<.01 for multiple testing*.*

^g^*R^2^*: percentage of variance explained by moderator.

^h^NNT: number needed to treat.

^i^Significant at *P*<.05.

^j^TAU: treatment as usual.

^k^One between-groups *P* value and R^2^ value are provided for each subgroup comparison, reported on the row of the first subgroup category.

^l^Where there are no comparisons within a subgroup, SMD, 95% confidence intervals and I^2^ values are not reported.

^m^ITT: intention to treat.

##### Assessment of Publication Bias

**Figure 5:**
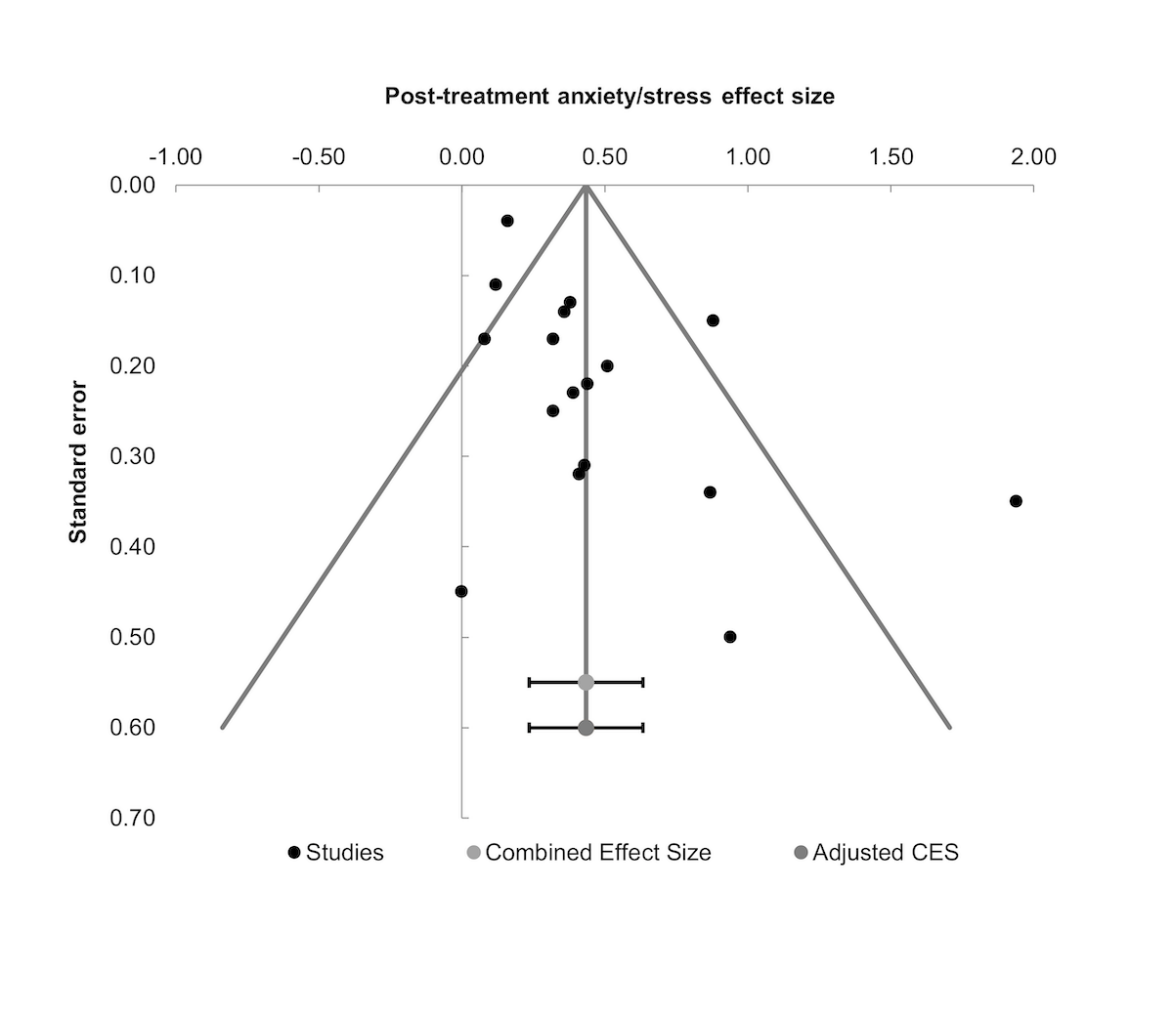
Funnel plot for distribution of studies reporting e-therapy versus controls post-treatment anxiety/stress outcomes.

Visual inspection of the funnel plot in Figure 5 suggested that there was some asymmetry in the distribution of studies reporting posttreatment anxiety and stress outcomes. However, the trim and fill imputation did not impute any missing data in relation to smaller studies in favor of controls or minimal differences between groups producing an adjusted effect size identical to the initial pooled SMD. The Egger regression failed to detect sufficient asymmetry in the study distribution of posttreatment anxiety and stress outcomes (B=–0.35; t_16_=1.82; *P*=.09). Taken together, the multiple assessments of publication bias imply a minimal-to-small influence of reporting bias on the overall e-therapy treatment effect for anxiety and stress outcomes. There were insufficient studies (*k*<10) to enable accurate assessment of publication bias on comparisons of follow-up anxiety and stress outcomes.

## Discussion

### Principal Findings

This study has been the first attempt to assess the breadth and quality of the evidence base for NHS-recommended e-therapies and to quantify the efficacy of this health technology through a meta-analysis of the clinical trial evidence base. Only 15% (7/48) of the NHS-recommended e-therapies had eligible RCT studies underpinning their clinical evaluation. Of the 7 e-therapies with RCT evidence, 2 contributed a single RCT study to the meta-analysis, and there was poor and variable reporting of version numbers across studies. These findings are at odds with the philosophy of evidenced-based practice, whereby clinical guidelines are underpinned by gold standard evidence of efficacy. Overall, however, the available good quality evidence shows that the e-therapies tested do benefit adult participants in better managing anxiety, stress and depression compared with controls, and this appears to be a durable effect in the short to medium term. The magnitude of the e-therapy treatment effects found here mirrors the effect sizes seen in the overall LI intervention evidence base (g=0.2-0.5) [5]. The NNT analysis suggests that for every 5 patients treated with an e-therapy, one has a good outcome. The acceptability and efficacy of the e-therapies without RCT evidence (ie, 85%, 41/48) of those actually recommended for use in the NHS) remains open to question. It would be premature to clinically champion any single e-therapy as being the most effective at this point in time. MoodGYM has been exposed to most evaluation and scrutiny, but it was unclear whether differing versions were being tested.

The acceptability of e-therapies can be called into question because of the higher dropout rates compared with controls reported here. Criticisms of LI psychological interventions, and e-therapies in particular, have been previously made concerning their high dropout rates being an index for poor patient acceptability, because of the low therapist contact and time approach [13,16,57,58]. Dropout rates may also have been influenced by multiple (unmeasured) factors such as the poor face validity of the CBT theoretical approach [59], low readiness to change, poor attitudes to the delivery of eHealth [60], and the usability or characteristics of the web or app design itself [61,62]. Ongoing issues with poor acceptability will remain an obstacle in the commissioning and delivery of e-therapies as frontline LI psychological interventions. Clearly, the clinical utility of any e-therapies needs to be considered in a matrix of cost, safety, acceptability, feasibility, and efficacy evidence [63].

Comparison of study characteristics highlighted noteworthy commonalities and differences across and between e-therapies. First, 5 of the 7 e-therapies evaluated were based on CBT (one other was based on CBT alongside other approaches). This mirrors that LI interventions as a whole tend to be based and focused on variants of CBT [64]. Recent innovations in e-therapies have included acceptance and commitment therapy [65], interpersonal psychotherapy [66], mindfulness [67], and psychodynamic psychotherapy [68]. Second, 6 of the 7 e-therapies were web based, so the clinical utility of smartphone-based app delivery of NHS-recommended e-therapies has not been appropriately empirically evaluated.

Variations in e-therapy treatment effects were explored with moderator analyses, as a previous individual participant meta-analysis of e-therapies for depression found few significant moderators [13]. Significantly larger e-therapy effects were apparent when compared with wait list controls (for posttreatment depression outcomes), for patients with greater baseline severity (for follow-up depression outcomes), and when there was a greater amount of therapist input (for end of treatment anxiety and stress outcomes). However, the effects of control type and amount of therapist input did not remain significant after accounting for multiple testing, so caution should be taken with any conclusions. Larger wait list comparison effects are commonly observed in psychotherapy trials and when taken in isolation can lead to overestimated treatment effects [69]. E-therapy effects shrunk as the activeness of comparators increased. In this review, baseline severity was only a significant moderator at follow-up. Greater e-therapy benefits for higher baseline depression severity have previously been shown to predict better outcomes for internet-based CBT [70]. The trend for e-therapies with a greater amount of therapist input generating better outcomes has been widely reported [71-73]. It is worth noting that e-therapy typologies in this meta-analysis emphasized some therapist contact, but that contact time was still relatively brief because of the LI approach. Furthermore, 75% (18/24 studies of 4 different apps) had less than 30 min of real-time person-to-person support. The efficacy of LI interventions appears to be better enabled when supported by even brief interpersonal contact [72,73].

### Limitations

This review has several limitations, which also highlight how the e-therapy evidence base could be further developed. First, although the included studies were restricted to high-quality RCT evidence, the GRADE approach highlighted issues with inconsistency across results, treatment comparisons, and some imprecision resulting in meta-analytic comparisons of moderate-to-low quality. Second, there are limitations concerning the generalizability of the findings. This review was limited to the treatment of depression, anxiety, and stress with e-therapies and so cannot comment on applicability to other clinical presentations. Services in the United Kingdom use the NICE guidelines to organize the delivery of treatments for anxiety and depression via stepped-care principles. Therefore, the generalizability of results from this meta-analysis is less applicable for different approaches to mental health delivery, for example, via stratified care [74]. The inclusion of only those e-therapies recommended by the NHS excluded those e-therapies very similar in technical format and content.

Third, there were some methodological weaknesses that may have introduced bias, and the conclusions should be treated with caution. The lack of formal screening and selection of articles by a second reviewer is a major limitation that may have led to bias in terms of which studies were selected for inclusion and therefore influenced the results. Similarly, the quality ratings of the studies were made by raters that were not independent from the meta-analysis, and levels of agreement were not optimal [75]. In addition, restrictions in the search strategy may have missed eligible studies or excluded studies evaluating an NHS e-therapy for other clinical presentations or outcomes [76]. Given that eHealth is a rapidly expanding area that makes reviews outdated relatively quickly, the duration since the final searches were conducted (April 2019) means there will undoubtedly be additional relevant e-therapy trials now available. Since the final searches, trials of 3 NHS e-therapies (all with existing trial evidence) have been published; an RCT of SilverCloud used in IAPT [77], evaluations of MoodGYM [78], and Headspace in student samples [79,80].

Finally, synthesis and analysis were restricted by the data from the available studies. The number of trials conducted was small, and thus restricted the power and range of possible moderator analyses. The original studies had the common methodological flaws of limited diagnostic assessments of participants, inconsistent reporting of e-therapy version numbers, overuse of self-reported measures rather than independent assessment, lack of reporting of adverse event rates [63], lack of measures of e-therapy adherence, and lack of true long-term follow-up. The frequent use of passive controls risked inflating treatment effect sizes in meta-analyses [81], and there were insufficient active comparators to establish efficacy of e-therapies vs other therapies. There was no standard definition of dropout or treatment completion across the studies, and therefore, we were forced to adopt the definition used by each study. It is acknowledged that dropout is a limited proxy for acceptability [82] and that wider indices of acceptability also include understanding barriers to e-therapy engagement.

### Research and Service Implications

Finding studies relating to a specific e-therapy by searching for its name in academic databases proved difficult. This was because before commercialization, many e-therapy platforms were known by their initial project name and not their eventual product name. A solution to this problem would be to ensure that e-therapy developers and researchers register their software on a public database with a unique identifier to be referenced in any subsequent publications. Trials of e-therapies should also be reported according to the CONSORT-EHEALTH checklist [56], and the e-therapy version should be indicated using semantic versioning to clarify whether the e-therapy program being evaluated has been updated (ie, reporting the major, minor, and patch version [eg, version 2.1.1]).

Several e-therapies included in this review were developed to be available without clinical support or guidance (eg, MoodGYM and Headspace). Given that e-therapies outperform controls (with moderate effects compared with wait list), e-therapies may offer particular promise as a waitlist intervention. Although unguided e-therapy may be beneficial to patients waiting for face-to-face psychological interventions, the trend observed in this review and findings from previous studies imply that some clinician involvement is important for ensuring good outcomes if an e-therapy is the sole intervention [72,73]. The manner in which e-therapies can be effectively blended with face-to-face psychological therapies is currently poorly understood and demands more research. Studies also need to be conducted on the utility of e-therapies as wait list interventions.

Given the recent availability of differing theoretical approaches, patient choice for e-therapy can now be offered and researched. Treatment completion rates need to be consistently reported, and trials adopt the ITT approach to reduce biasing treatment effects. Consistent reporting of safety issues (eg, via untoward incident rates) is needed for e-therapies. Health economic evaluations that are embedded in clinical trials need to be increased. A dropout meta-analysis (with independent study quality ratings of all studies using the latest version of the Cochrane risk of bias tool) of this evidence base is now also indicated to better index e-therapy acceptability issues [83]. Little is known about why patients’ drop out of e-therapies, and qualitative investigations would be useful here. Treatment adherence (ie, how much time is spent and how many modules of eHealth are completed by participants) needs to be more consistently reported. The role of moderating factors of treatment outcome in e-therapies needs to be better researched, particularly the role of variables such as blended vs pure e-therapy approaches, time spent on the app, and theoretical approach. E-therapies potentially still play an important role in clinical services, regardless of the organizational system used to coordinate delivery of care [84], particularly when the approach has been well evaluated.

### Conclusions

In this meta-analysis of *gold standard* clinical trials, e-therapies have been found to be efficacious as LI psychological interventions that produce small beneficial effects for adults with depression, anxiety, and stress compared with controls. However, only a relatively small proportion of NHS-recommended e-therapies had been subjected to such *gold standard* evaluation. Although these conclusions should be considered in light of the methodological limitations, the targeted nature of this review to NHS-recommended e-therapies still has relevance to the global field of e-therapies. This is particularly through highlighting the need to consistently integrate high quality and controlled evaluation into the technological development of e-therapies. This is to ensure eventual safe and evidence-based e-therapy practice in routine clinical services. Technological development and scrupulous evaluation of e-therapies need to be conducted in parallel and considered in equipoise.

## Appendix

Multimedia Appendix 1 Example search strategy.

Multimedia Appendix 2 Characteristics of included studies.

Multimedia Appendix 3 Summary of e-therapy version numbers.

Multimedia Appendix 4 Sensitivity analyses.

## Abbreviations

GP: general practitioner
IAPT: Improving Access to Psychological Therapies
ITT: intention to treat
LI: low intensity
NHS: National Health Service
NICE: National Institute for Health and Care Excellence
NNT: number needed to treat
RCT: randomized controlled trial
SMD: standardized mean difference
TAU: treatment as usual
