# Supplementary material for: Acceptability and Effectiveness of NHS-Recommended e-Therapies for Depression, Anxiety, and Stress: Meta-Analysis
Source: J Med Internet Res. 2020 Oct 28;22(10):e17049. doi: 10.2196/17049 (PMC7657731; doi:10.2196/17049)
Supplement: Multimedia Appendix 1 [file jmir_v22i10e17049_app1.pdf]

Multimedia Appendix 1: Example Search Strategy

Electronic searches were last conducted on the following databases: PsycINFO, Web of Science, PubMed on the 11th of April 2019.

FULL SEARCH STRATEGY FOR PUBMED

"Living Life to the Full" AND Website
"MoodGym" AND Website
"Big White Wall" AND Website
"Beating the Blues" AND Website
"SilverCloud Health" AND Website
"Ieso Digital Health Ltd" AND Website
"Fear Fighter" AND Website
"HeadSpace" AND App
"Buddy App" AND Website
"Don't Panic!" AND App
"MyMoodTracker" AND App
"Mindfulness Bell" AND App
"Moodkit – Mood Improvement Tools" AND App
"Thought Diary Pro" AND App
"WellMind" AND App
"Moodometer" AND App
"Kooth" AND Website
"CBTReferee" AND App
"iCBT" AND App
"Thought Diary" AND App
"Stay Alive" AND App
"Take a break!" AND App
"Mindshift" AND App
"Moodscope" AND Website
"DigitalMeds" AND App
"How Are You App" AND App
"Mindfulness by Digipill" AND App
"Mindlogr" AND App
"Panic Attack Aid" AND App
"Phobia Free" AND App
"Stress Management App" AND App
"WorkGuru" AND Website
"Worry Watch" AND App
"MindEd" AND App
"Puffell" AND Website
"Virtual Hope Box" AND App
"Aventurine Mood Improver" AND App
"Black Rainbow" AND App
"Depression Calculator" AND App
"Five Ways to Wellbeing" AND App
"Ginsberg" AND App
"Happy Healthy App" AND App
"HealthStored" AND App
"Healthy Living" AND App
"Hello Brain Health" AND App
"Moodbug" AND App
"SAM: Self-help for Anxiety" AND App
"Stress & Anxiety Companion" AND App
