# Supplementary material for: Acceptability and Effectiveness of NHS-Recommended e-Therapies for Depression, Anxiety, and Stress: Meta-Analysis
Source: J Med Internet Res. 2020 Oct 28;22(10):e17049. doi: 10.2196/17049 (PMC7657731; doi:10.2196/17049)
Supplement: Multimedia Appendix 2 [file jmir_v22i10e17049_app2.pdf]

Multimedia Appendix 2: Characteristics of included studies

Table A1. Characteristics of included studies.

**Study, authors (year)**	**Presenting problem**	**Age (years), mean (SD)**	**Sex (male), %**	**Treatment conditions (if applicable, TAU or placebo description)**	**Population, N**	**E-therapy format and duration[a]**	**Follow-up (months)**	**Drop out, n (%)[b]**
Proudfoot et al. (2003) [32]	Anx or Dep[c]	44.63 (14.41)	26.35	• Beating the Blues<br>• TAU[d] (usual GP[e] care including medication, practical or social help, referral to mental health professional or physical investigation)	• 89<br>• 78	PSH[f]; 8×50 min sessions, weekly	1, 3, and 6	• 39 (44)<br>• 27 (35)
Grime (2004) [33]	Anx or Dep or Stress	39 (9.74)	41.66	• Beating the Blues<br>• TAU (conventional care including medication, counselling, and other care)	• 24<br>• 24	PSH; 8 sessions, weekly	1, 3, and 6	• 5 (21)<br>• 1 (4)
Proudfoot et al. (2004) [34]	Anx or Dep	43.51 (13.91)	26.28	• Beating the Blues<br>• TAU (usual GP care including medication, practical or social help, referral to mental health professional or physical investigation)	• 146<br>• 128	PSH; 8×50 min sessions, weekly	1, 3, and 6	• 54 (37)<br>• 35 (27)
Marks et al. (2004) [35]	Panic or phobia	38.13 (12.42)	31	• FearFighter<br>• Placebo (online self-relaxation de-STRESS)	• 37<br>• 17	MCT[h]; 6×60 min sessions over 10 weeks	1	• 16 (43)<br>• 1 (6)

Schneider et al. (2005) [36]	Panic or phobia	39 (11)	26.47	• FearFighter<br>• Placebo (online self-relaxation Managing Anxiety)	• 45<br>• 23	MCT; 6 sessions over 10 weeks	1	• 10 (22)<br>• 6 (26)
Mackinnon et al. (2008) [37]	Dep	36.8 (9.3)	27.54	• MoodGYM<br>• Placebo (Bluepages psychoeducation)<br>• Placebo (attention-control structured phone calls)	• 182<br>• 165<br>• 178	PSH; 5 sessions, weekly	6 and 12	• 46 (25)<br>• 29 (18)<br>• 21 (12)
Kessler et al. (2009) [38]	Dep	34.95 (11.60)	31.99	• Ieso<br>• TAU + wait list for online CBT (usual GP care including medication, referral for non-CBT psychotherapy)	• 149<br>• 148	PTD[i]; 10×55 min sessions within 16 weeks	4	• 71 (48)<br>• 11 (8)
Ellis et al. (2011) [39]	Anx or Dep	19.67 (1.66)	23	• MoodGYM<br>• Placebo (online peer support group MoodGarden)<br>• Wait list	• 13<br>• 13<br>• 13	PSH; 5 × 60 min sessions, weekly	None	• NR[j]<br>• NR<br>• NR
Farrer et al. (2011) [40]	Dep	41.53 (12.36)	18.06	• MoodGYM<br>• MoodGYM & tracking phone calls<br>• Placebo (attention-control tracking phone calls)<br>• No treatment	• 38<br>• 45<br>• 37<br>• 35	PSH; 5 sessions over 6 weeks	6	• 11 (29)<br>• 25 (56)<br>• 4 (11)<br>• 8 (23)
Høifødt et al.	Dep	37.18	27.36	• MoodGYM	• 52	MCT; 6×45-60 min	6	• 19 (36)

(2013) [41]		(11.09)		• Wait list	• 54	sessions, weekly		• 7 (13)
Lintvedt et al. (2013) [42]	Distress	28.15 (7.36)	23.31	• MoodGYM<br>• Wait list	• 81<br>• 82	SAT[g]; 5 sessions, weekly	None	• 38 (47)<br>• 23 (28)
Powell et al. (2013) [43]	No criteria	41.14 (13.01)	22.12	• MoodGYM<br>• Wait list	• 1534<br>• 1536	SAT; 5 sessions, weekly	1.5	• 977 (64)<br>• 317 (21)
Sethi (2013) [44]	Anx or Dep	20.13 (1.39)	30.44	• MoodGYM<br>• No treatment	• 23<br>• 23	PSH; 5×60 min sessions, weekly	None	• 0 (0)<br>• 0 (0)
Howells et al. (2016) [45]	No criteria	40.3 (10.54)	11.86	• Headspace<br>• Placebo (Catch notes attention-control task)	• 97<br>• 97	SAT; 10×10 min sessions, daily	None	• 27 (28)<br>• 22 (23)
Phillips et al. (2014) [46]	Dep	42.45 (9.6)	47.44	1. MoodGYM<br>2. Placebo (websites of mental health info)	• 318<br>• 319	PSH; 5×60 min sessions, weekly	1.5	• 154 (48)<br>• 143 (45)
Twomey et al. (2014) [47]	Anx or Dep or Stress	33.75 (10.01)	22.73	• MoodGYM<br>• Wait list	• 101<br>• 100	SAT; 5×20-40 min sessions , weekly	3 (insufficient data available)	• 45 (56)<br>• 20 (29)
Gilbody et al. (2015) [48]	Dep	39.87 (12.65)	33.14	• Beating the Blues<br>• MoodGYM<br>• TAU (usual GP prescribed care including medication, counselling, psychological services, or secondary care)	• 210<br>• 242<br>• 239	• PSH; 8 × 50 min sessions, weekly<br>• SAT; 6 × 50 min sessions, weekly	8 and 20	• 45 (21)<br>• 60 (25)<br>• 60 (25)
Richards et al. (2015)	Dep	39.86 (10.94)	27.13	• SilverCloud Health<br>• Wait list	• 133<br>• 129	MCT; 8 sessions, weekly	6	• 73 (55)<br>• 37 (29)

[49]								
Richards et al. (2016) [50]	Anx	23.82 (7.05)	22.6	• SilverCloud Health<br>• Wait list	• 70<br>• 67	MCT; 6 sessions, weekly	None	• 11 (16)<br>• 14 (21)
Carolan et al. (2017) [51]	Stress	41.0 (10.2)	15	• WorkGuru<br>• Wait list	• 56<br>• 28	PSH; 7 core and 3 additional modules, 60 min per week for 8 weeks	2	• 17 (30)<br>• 3 (11)
Flett et al. (2018) [52]	Not specified	20.08 (2.88)	29.8	• Headspace<br>• Placebo (Attention-control Evernote organization app)	• 72<br>• 75	SAT; 10 sessions, daily for 10 days (discretional use for next 30 days until follow-up)	1	• 5 (7)<br>• 8 (11)
Forand et al. (2018) [53]	Dep	32.85 (11.75)	23.85	• Beating the Blues<br>• Wait list	• 60<br>• 30	PSH[h]; 8×45-60 min sessions, weekly	None	• 17 (29)<br>• 3 (10)
Bostock et al. (2019) [54]	Stress	35.5 (7.6)	40.76	• Headspace<br>• Wait list	• 128<br>• 110	SAT; 45×10-20 min session daily for 45 days	2 (e-therapy group only)	• 5 (4)<br>• 4 (4)
Löbner et al. (2019) [55]	Dep[c]	43.85 (13.7)	31.53	• MoodGYM<br>• TAU (usual GP[e] care including medication, referral for psychotherapy or psychiatric inpatient or outpatient care)	• 320<br>• 327	SAT; 5 sessions, weekly	6	• 53 (17)<br>• 20 (6)

[a]Self-help typology coded according to the framework by Newman et al [19].
[b]Dropout rate based on percentage of noncompleters, as reported by the study.
[c]Anx or dep: anxiety or depression.
[d]TAU: treatment as usual.
[e]GP: general practitioner.
[f]PSH: predominantly self-help.
[g]SAT: self-administered therapy.
[h]MCT: minimal contact therapy.
[i]PTD: predominantly therapist delivered.
[j]NR: not reported.

References cited in the table.

32. Proudfoot J, Goldberg D, Mann A, Everitt B, Marks I, Gray JA. Computerized, interactive, multimedia cognitive-behavioural program for anxiety and depression in general practice. Psychol Med 2003;33(2):217–227. PMID:12622301
33. Grime PR. Computerized cognitive behavioural therapy at work: A randomized controlled trial in employees with recent stress-related absenteeism. Occup Med (Chic Ill) 2004;54(5):353–359. PMID:15289593
34. Proudfoot J, Ryden C, Everitt B, Shapiro DA, Goldberg D, Mann A, Tylee A, Marks I, Gray JA. Clinical efficacy of computerised cognitive-behavioural therapy for anxiety and depression in primary care: Randomised controlled trial. Br J Psychiatrysychiatry [Internet] 2004;185:46–54. PMID:15231555
35. Marks IM, Kenwright M, McDonough M, Whittaker M, Mataix-Cols D. Saving clinicians' time by delegating routine aspects of therapy to a computer: A randomized controlled trial in phobia/panic disorder. Psychol Med 2004;34(1):9–17. PMID:14971623
36. Schneider AJ, Mataix-Cols D, Marks IM, Bachofen M. Internet-guided self-help with or without exposure therapy for phobic and panic disorders: A randomised controlled trial. Psychother Psychosom 2005;74(3):154–164. PMID:15832066

37. Mackinnon A, Griffiths KM, Christensen H. Comparative randomised trial of online cognitive-behavioural therapy and an information website for depression: 12-Month outcomes. Br J Psychiatry 2008;192(2):130–134. PMID:18245031

38. Kessler D, Lewis G, Kaur S, Wiles N, King M, Weich S, Sharp DJ, Araya R, Hollinghurst S, Peters TJ. Therapist-delivered Internet psychotherapy for depression in primary care: a randomised controlled trial. Lancet Elsevier Ltd; 2009 Aug;374(9690):628–34. PMID:19700005

39. Ellis L, Campbell A, Sethi S, O'Dea B. Comparative randomized trial of an online cognitive-behavioral therapy program and an online support group for depression and anxiety. J cybertherapy Rehabil 2011;4(4):461–467.

40. Farrer L, Christensen H, Griffiths KM, Mackinnon A. Internet-based CBT for depression with and without telephone tracking in a national helpline: Randomised controlled trial. PLoS One 2011;6(11). PMID:22140514

41. Høifødt RS, Lillevoll KR, Griffiths KM, Wilsgaard T, Eisemann M, Waterloo K, Kolstrup N. The clinical effectiveness of web-based cognitive behavioral therapy with face-to-face therapist support for depressed primary care patients: Randomized controlled trial. J Med Internet Res 2013;15(8). PMID:23916965

42. Lintvedt OK, Griffiths KM, Sørensen K, Østvik AR, Wang CEA, Eisemann M, Waterloo K. Evaluating the effectiveness and efficacy of unguided internet-based self-help intervention for the prevention of depression: A randomized controlled trial. Clin Psychol Psychother 2013;20(1):10–27. PMID:21887811

43. Powell J, Hamborg T, Stallard N, Burls A, Mcsorley J, Bennett K, Griffiths KM, Christensen H. Effectiveness of a web-based cognitive-behavioral tool to improve mental well-being in the general population: Randomized controlled trial. J Med Internet Res 2013;15(1):1–17. PMID:23302475

44. Sethi S. Treating youth depression and anxiety: A randomised controlled trial examining the efficacy of computerised versus face-to-face cognitive behaviour therapy. Aust Psychol 2013;48(4):249–257. [doi: 10.1111/ap.12006]

45. Howells A, Ivtzan I, Eiroa-Orosa FJ. Putting the 'app' in Happiness: A Randomised Controlled Trial of a Smartphone-Based Mindfulness Intervention to Enhance Wellbeing. J Happiness Stud 2016;17(1):163–185. [doi: 10.1007/s10902-014-9589-1]

46. Phillips R, Schneider J, Molosankwe I, Leese M, Foroushani PS, Grime P, McCrone P, Morriss R, Thornicroft G. Randomized controlled trial of computerized cognitive behavioural therapy for depressive symptoms: Effectiveness and costs of a workplace intervention. Psychol Med 2014;44(4):741–752. PMID:23795621

47. Twomey C, O'Reilly G, Byrne M, Bury M, White A, Kissane S, McMahon A, Clancy N. A randomized controlled trial of the computerized CBT programme, MoodGYM, for public mental health service users waiting for interventions. Br J Clin Psychol 2014;53(4):433–450. PMID:24831119

48. Gilbody S, Littlewood E, Hewitt C, Brierley G, Tharmanathan P, Araya R, Barkham M, Bower P, Cooper C, Gask L, Kessler D, Lester H, Lovell K, Parry G, Richards DA, Andersen P, Brabyn S, Knowles S, Shepherd C, Tallon D, White D. Computerised cognitive behaviour therapy (cCBT) as treatment for depression in primary care (REEACT trial): Large scale pragmatic randomised controlled trial. BMJ 2015;351:1–13. PMID:26559241

49. Richards D, Timulak L, O'Brien E, Hayes C, Vigano N, Sharry J, Doherty G. A randomized controlled trial of an internet-delivered treatment: Its potential as a low-intensity community intervention for adults with symptoms of depression. Behav Res Ther [Internet] Elsevier Ltd; 2015;75:20–31. PMID:26523885

50. Richards D, Timulak L, Rashleigh C, McLoughlin O, Colla A, Joyce C, Doherty G, Sharry J, Duffy D, Anderson-Gibbons M. Effectiveness of an internet-delivered intervention for generalized anxiety disorder in routine care: A randomised controlled trial in a student population. Internet Interv [Internet] The Authors; 2016;6:80–88. PMID:30135817

51. Carolan S, Harris PR, Greenwood K, Cavanagh K. Increasing engagement with an occupational digital stress management program through the use of an online facilitated discussion group: Results of a pilot randomised controlled trial. Internet Interv [Internet] Elsevier; 2017;10(August):1–11. PMID:30135747

52. Flett JAM, Hayne H, Riordan BC, Thompson LM, Conner TS. Mobile mindfulness meditation: A randomised controlled trial of the effect of two popular apps on mental health. Mindfulness (N Y) Mindfulness; 2019;10(5):863–876. [doi: 10.1007/s12671-018-1050-9]

53. Forand NR, Barnett JG, Strunk DR, Hindiyeh MU, Feinberg JE, Keefe JR. Efficacy of guided iCBT for depression and mediation of change by cognitive skill acquisition. Behav Ther [Internet] Elsevier Ltd; 2018;49(2):295–307. PMID:29530267

54. Bostock S, Crosswell AD, Prather AA, Steptoe A. Mindfulness on-the-go: Effects of a mindfulness meditation app on work stress and well-being. J Occup Health Psychol 2019;24(1):127–138. PMID:29723001

55. Löbner M, Pabst A, Stein J, Dorow M, Matschinger H, Luppa M, Maroß A, Kersting A, König HH, Riedel-Heller SG. Computerized cognitive behavior therapy for patients with mild to moderately severe depression in primary care: A pragmatic cluster randomized controlled trial (@ktiv). J Affect Disord 2018;238(June):317–326. PMID:29902736
