# Supplementary material for: Acceptability and Effectiveness of NHS-Recommended e-Therapies for Depression, Anxiety, and Stress: Meta-Analysis
Source: J Med Internet Res. 2020 Oct 28;22(10):e17049. doi: 10.2196/17049 (PMC7657731; doi:10.2196/17049)
Supplement: Multimedia Appendix 3 [file jmir_v22i10e17049_app3.pdf]

Multimedia Appendix 3: Summary of E-therapy Version Numbers

Table A2. Summary of version numbers for e-therapies implemented in the included studies.

**Paper**	**Software**	**Version**	**CONSORT-EHEALTH**	**Comments**
(Proudfoot et al. 2003)	Beating the blues	No Version given.<br><br>See comments.	Predates the checklist publication	Version not stated but reference to the following paper given:<br><br>Gray, J. A., Proudfoot, J. & Swain, S. (2000). 'Beating the Blues': computerised cognitive-behavioural therapy. Journal of Primary Care Mental Health 4, 17–18.<br><br>(could be v1.0 see below)
(Proudfoot et al. 2004)	Beating the blues	No Version given.<br><br>However, it is v1.0 see comments.	Predates the checklist publication	Version not stated but paper gives indication that this version is the same as the one used in the previous 2003 study.<br><br>*"In a previous study (Proudfoot et al, 2003) we established the efficacy of an eight-session interactive, multimedia Computerised cognitive–behavioural therapy package, Beating the Blues. In an expanded data-set we now investigate interactions of this ther- apy with clinical, demographic and setting demographic variables, and again demonstrate its efficacy."*<br><br>(Forand et al. 2018) **indicates this paper used version v1.0** and featured:<br>• British actors and British English content<br>• Between-session projects printed on paper<br>• Sessions had to be completed in one sitting<br>• Focused on clinical depression and anxiety<br><br>It could also be assumed that (Proudfoot et al. 2003) used v1.0 based on the above info.

(Grime 2004)	Beating the blues	No Version given.	Predates the checklist publication	Could not determine version based on information in paper.
(Gilbody et al. 2015)	Beating the blues	No Version given.	No Checklist	Could not determine version based on information in paper.
(Forand et al. 2018)	Beating the blues	US v2.5	No Checklist	United States v2.5 licensed by U2 Interactive, LLC<br><br>It includes a number of differences to v1.0:<br><br>• American actors and American English content<br>• Between-session projects completed online or printed<br>• Participants could leave the session and re-enter at the same point<br>• Focused stress, tension, anxiety, and depression<br>• Content re-ordered and modified, user interface updated, and artwork updated and modernized
(Marks et al. 2004)	FearFighter	No Version given.	Predates the checklist publication	Could not determine version based on information in paper.
(Schneider et al. 2005)	FearFighter	No Version given.	Predates the checklist publication	Could not determine version based on information in paper.
(Howells, Ivtzan, and Eiroa-Orosa 2016)	HeadSpace	No Version given.<br><br>See comments.	No Checklist	Could not determine version based on information in paper.<br><br>However, there is a little bit of evidence we can use to give a rough estimate:<br><br>Headspace was launched in 2012: https://web.archive.org/web/20120110193741/http://www.getsomeheadspace.com/News/headspace-blog/the-launch-of-the-headspace-journey.aspx<br><br>Version 2.0 was released in 2014: https://www.fastcompany.com/3041402/the-monk-and-the-mad-man-making-mindfulness-for-the-masses

				Version 3.0 was release in 2017 based on APK history: https://www.apk4fun.com/history/13427/
(Flett et al. 2019)	HeadSpace	No Version given.	No Checklist	Could not determine version based on information in paper.
(Bostock et al. 2019)	HeadSpace	No Version given.	No Checklist	Could not determine version based on information in paper.
(Kessler et al. 2009)	IESO	No Version given.	Predates the checklist publication	Reference is given to the organisation "PsychologyOnline" in this paper. This is paper is pre the name change to IESO.
(Mackinnon, Griffiths, and Christensen 2008)	MoodGYM	No Version given.	Predates the checklist publication	Could not determine version based on information in paper.
(Ellis et al. 2011)	MoodGYM	No Version given.	Predates the checklist publication	Could not determine version based on information in paper.
(Farrer et al. 2011)	MoodGYM	No Version given.	Predates the checklist publication	Could not determine version based on information in paper.
(Powell et al. 2013)	MoodGYM	No Version given.<br><br>See comments.	CONSORT-EHEALTH checklist V.16	English version<br><br>"We made slight modifications to some phrases used in the MoodGYM tool to replace Australian colloquialisms with their English equivalent. We added logos to indicate affiliation to the NHS and University of Warwick (lead academic institution)."<br><br>Information drawn from CONSORT-EHEALTH checklist V.16 included with the paper states for subitem 5-iii<br><br>"The MoodGYM intervention us well established and previous work detailing the intervention has been published in JMIR and elsewhere (and we cite this)."
(Lintvedt et al. 2013)	MoodGYM	Mark III	No checklist	Norwegian version

				"The content of both websites was translated into Norwegian at the Department of Psychology, University of Tromsø, in collaboration with the Australian National University (ANU, 2010a, 2010b)."
(Høifødt et al. 2013)	MoodGYM	Version III	CONSORT-EHEALTH checklist V1.6.2 but referenced but unavailable.	Norwegian version<br><br>The paper refers to a CONSORT-EHEALTH checklist V1.6.2 as a multimedia appendix but this is unavailable.
(Sethi 2013)	MoodGYM	No Version given.	No checklist	Could not determine version based on information in paper.
(Phillips et al. 2014)	MoodGYM	No Version given.<br><br>See comments.	No checklist	Reference is given to ANU (2012). MoodGYM: Welcome (https://://moodgym.anu. edu.au/welcome). Australia National University. Accessed 3 December 2012.<br><br>If we use the internet archive to look this url up during the time period, we find Version III.<br><br>https://web.archive.org/web/20121018220901/https://moodgym.anu.edu.a u/welcome
(Twomey et al. 2014)	MoodGYM	No Version given.	No checklist	Could not determine version based on information in paper.
(Löbner et al. 2018)	MoodGYM	Version III	No checklist	German version<br><br>The German version of moodgym was developed by different specialists in mental health care including psychotherapists and psychiatrists at ISAP. Adaptions during the translation process were included to account for German cultural norms. During the trial, German moodgym (version III) was exclusively available for study participants in the in- tervention group.

(D. Richards et al. 2015)	SilverCloud	No Version given.<br><br>See comments.	No checklist	The program is called “SilverCloud Space from Depression program” in this paper.<br><br>Modules:<br>• Getting started<br>• Tune In I: getting to grips with mood<br>• Tune in II: spotting thoughts<br>• Change It I: boosting behavior<br>• Change It III: core beliefs<br>• Bringing It all together
(Derek Richards et al. 2016)	SilverCloud	No Version given.<br><br>See comments.	No checklist	The program is called “The Calming Anxiety supported programme” in this paper. I note Module 1 is called “SilverCloud”.<br><br>Modules:<br>• Getting Started<br>• Understanding Moods and Emotions<br>• Anxious thoughts and worry<br>• Face your Anxiety, Step by Step<br>• Challenge your Anxious Thoughts<br>• Bringing it all Together
(Carolan et al. 2017)	WorkGuru	No Version given.	Checklist completed (version not indicated). Not accessible.	Could not determine version based on information in paper.<br><br>The paper refers to a CONSORT-EHEALTH checklist, but this is not included as an appendix with the paper.
