# Supplementary material for: Acceptability and Effectiveness of NHS-Recommended e-Therapies for Depression, Anxiety, and Stress: Meta-Analysis
Source: J Med Internet Res. 2020 Oct 28;22(10):e17049. doi: 10.2196/17049 (PMC7657731; doi:10.2196/17049)
Supplement: Multimedia Appendix 4 [file jmir_v22i10e17049_app4.pdf]

Multimedia Appendix 4: Sensitivity Analyses

Outliers

Table A3. Sensitivity analyses of effect E-therapy versus controls on depression and anxiety/stress outcomes with outliers removed.

	Main analysis	Sensitivity analysis						
	SMD (g)	*k*	*n*	SMD (g)[a]	95% CI	*P*	I^2 (%)[b] [I^2 95% CI]	NNT
**Depression**								
Post-treatment[c]	0.38	25	6986	0.34	0.23 to 0.44	<.001*	63%* [43% to 76%]	5.26
Follow-up[d]	0.25	15	5667	0.22	0.08 to 0.36	.001*	64%* [37% to 79%]	8.09
**Anxiety/stress**								
Post-treatment[e]	0.43	16	4817	0.37	0.23 to 0.50	<.001*	58% [27% to 76%]	4.85
Follow-up[f]	0.23	9	3939	0.22	0.18 to 0.26	<.001*	0% [0% to 0%]	8.09

Abbreviations: k = number of comparisons; *n* = number of participants; SMD = standardised mean difference; *g* = Hedges G; CI = confidence interval; NNT = number needed to treat.
[a] Positive effect size indicates in favour of e-therapy.
[b] Significance of associated *Q* statistic.
[c] Outlier Forrand (2018) removed.
[d] Outlier Farrer (2011) vs. no treatment removed.
[e] Outlier Sethi (2013) removed.
[f] Outlier Schneider (2005) removed.
*significant at $P < .05$ threshold.

Follow-up effects

Table A4. Sensitivity analyses of effect E-therapy versus controls on depression and anxiety/stress outcomes at short-, medium- and long-term follow-up.

	k	*n*	SMD (g)[a]	95% CI	*P*	I^2 (%)[b] [I^2 95% CI]	NNT
**Depression**							
Short-term follow-up (1-2 months)	7	3804	0.22	0.10 to 0.34	<.001*	9% [0% to 73%]	8.08

	Medium-term follow-up (4-6 months)	10	1710	0.40	0.17 to 0.62	.001*	59%* [17% to 80%]	4.49
	Long-term follow-up (8-12 months)	4	819	-0.08	-0.37 to 0.21	.38	24% [0% to 88%]	22.16[c]
**Anxiety/stress**								
	Short-term follow-up (1-2 months)	9	3873	0.26	0.13 to 0.39	<.001*	23% [0% to 64%]	6.85
	Medium-term follow-up (6 months)	4	399	0.24	0.13 to 0.35	<.001*	0% [0% to 0%]	7.42

Abbreviations: k = number of comparisons; n = number of participants; SMD = standardised mean difference; g = Hedges G; CI = confidence interval; NNT = number needed to treat.
[a] Positive effect size indicates in favour of e-therapy.
[b] Significance of associated Q statistic.
[c] Due to negative ES values indicates NNT to get one additional beneficial outcome for the control condition compared to e-therapy.
*significant at $P < .05$ threshold.
